# The relationship between components of hypoglycemia worries and avoiding hypoglycemia behavior in type 2 diabetes mellitus with hypoglycemia: a network analysis

**DOI:** 10.1186/s12888-023-04698-9

**Published:** 2023-03-28

**Authors:** Chao Wu, Wenwen Wang, Sizhe Cheng, Hongli Zhang, Lu Li, Ci Tian, Linyuan Zhang, Nana Chen, Juan Du, Lei Ren, Hongjuan Lang

**Affiliations:** 1 Nursing Department, Air Force Medical University, No.169 Changle West Road, Xi’an, Shaanxi 710032 China; 2 Department of Medical Statistics, School of Preventive Medicine, Air Force Medical University, Xi’an, China; 3 Department of Military Medical Psychology, Air Force Medical University, No.169 Changle West Road, Xi’an, Shaanxi 710032 China; 4 Department of Anesthesia Intensive Care Unit, Tangdu Hospital of Air Force Military Medical University, Xi′an, Shaanxi China; 5 Cardio-Thoracic Surgery, The 305th Hospital of the Chinese People’s Liberation Army, Beijing, China; 6 Department of Otolaryngology, Army Hospital of the Seventy-seventh Group, Jiajiang, Sichuan China

**Keywords:** T2DM patients, Hypoglycemia fear, Hypoglycemia worries, Avoiding hypoglycemia behavior, Network analyses

## Abstract

**Background:**

The fear of hypoglycemia in type 2 diabetes mellitus (T2DM) patients with hypoglycemia has seriously affected their quality of life. They are always afraid of hypoglycemia and often take excessive action to avoid it. Yet, researchers have investigated the relationship between hypoglycemia worries and excessive avoiding hypoglycemia behavior using total scores on self-report measures. However, network analysis studies of hypoglycemia worries and excessive avoiding hypoglycemia behavior in T2DM patients with hypoglycemia are lacking.

**Purpose:**

The present study investigated the network structure of hypoglycemia worries and avoiding hypoglycemia behavior in T2DM patients with hypoglycemia and aimed to identify bridge items to help them correctly treat hypoglycemia and properly deal with hypoglycemia fear.

**Methods:**

A total of 283 T2DM patients with hypoglycemia were enrolled in our study. Hypoglycemia worries and avoiding hypoglycemia behavior were evaluated with the Hypoglycemia Fear Scale. Network analyses were used for the statistical analysis.

**Results:**

B9 “Had to stay at home for fear of hypoglycemia” and W12 “I am worried that hypoglycemia will affect my judgment” have the highest expected influences in the present network. In the community of hypoglycemia worries, W17 “I worry about hypoglycemia during sleep” has the highest bridge expected influence. And in the community of avoiding hypoglycemia behavior, B9 “Had to stay at home for fear of hypoglycemia” has the highest bridge expected influence.

**Conclusion:**

Complex patterns of associations existed in the relationship between hypoglycemia worries and avoiding hypoglycemia behavior in T2DM patients with hypoglycemia. From the perspective of network analysis, B9 “Had to stay at home for fear of hypoglycemia” and W12 “I am worried that hypoglycemia will affect my judgment” have the highest expected influence, indicating their highest importance in the network. W17 “I worry about hypoglycemia during sleep” aspect of hypoglycemia worries and B9 “Had to stay at home for fear of hypoglycemia” aspect of avoiding hypoglycemia behavior have the highest bridge expected influence, indicating they have the strongest connections with each community. These results have important implications for clinical practice, which provided potential targets for interventions to reduce hypoglycemia fear and improve the quality of life in T2DM patients with hypoglycemia.

**Supplementary Information:**

The online version contains supplementary material available at 10.1186/s12888-023-04698-9.

## Introduction

Diabetes is characterized by hyperglycemia and can cause vascular and neurological damage, which seriously threatens the health of patients and contributed significantly to the global disease burden [1, 2]. As reported by the International Diabetes Federation, the prevalence of diabetes in the total population is 8.8% [3] and 415 million adults in the world had diabetes mellitus in 2015, which is projected to rise to 642 million by 2040. Type 2 diabetes mellitus (T2DM) accounts for over 90% of the total diabetes mellitus cases, with a prevalence of 11.6% among the Chinese adult population based on a recent large-scale population-based survey [4, 5] which has seriously affected people’s health and quality of life [6]. At the same time, with the change in lifestyle, it has been shown that the age of diagnosis of type 2 diabetes is gradually expanding and the trend of youth is obvious, which means that it will affect more people [7, 8].

Among the complications of type 2 diabetes, hypoglycemia is a common and potentially serious complication of diabetes [9, 10] which can lead to cognitive dysfunction, hemiplegia, blurred vision, decreased renal function, arrhythmia, coma, and even death [11]. The definition of type 2 diabetes mellitus with hypoglycemia is a glucose alert value of 3.9 mmol/L (70 mg/dL) or less. The frequencies of severe hypoglycemia in diabetes patients substantially vary across studies and a systematic review of 46 population-based studies estimated that was 6% [12], and the incidence of ordinary hypoglycemia was much higher. Hypoglycemia can cause dizziness, syncope, and even a sense of dying [13]. This negative emotional experience can lead patients to take “overcompensation behavior”, such as overly vigilant blood glucose monitoring, limiting physical activity, intentionally reducing the required drug dosage, or ingesting excessive carbohydrates to maintain blood glucose at a high level [14, 15]. It has become a major obstacle to the control of blood sugar, which can lead to the occurrence or aggravation of complications in patients and have a negative impact on the management of diabetes [16, 17].

There are many forms of hypoglycemia fears. ‘Hypoglycemia worries’ is an important aspect of hypoglycemia fears, which refers to the patient’s concern about hypoglycemia after experiencing it, and fear of developing again. Research shows that fear of hypoglycemia is closely related to worry, that is, the more fear of hypoglycemia, the more serious the anxiety of diabetes patients [18]. Besides worry, diabetes patients also have other negative emotions such as anxiety and depression, which seriously affect their subjective well-being [19, 20]. ‘Excessive avoiding hypoglycemia behavior’ is another important aspect of hypoglycemia fears [21]. In a survey of diabetes patients with hypoglycemia, results show that patients often take actions to maintain high blood sugar levels in order to avoid hypoglycemia again [22]. The behavior of avoiding hypoglycemia is not conducive to maintaining normal blood sugar of diabetes patients [23, 24]. Hypoglycemia worries and avoiding hypoglycemia behavior are two important aspects of hypoglycemic fears, one is ideological, the other is behavioral. However, previous studies investigating the relationship between hypoglycemia worries and avoiding hypoglycemia behavior have tended to consider hypoglycemia fear as a whole, such as T H Wieringa’s [25] research results shows that hypoglycemia is associated with lower quality of life in terms of hypoglycemia fear and diabetes symptom distress [25]. The determination of correlation based on the total score disguises the relationship between different dimensions of variables. The traditional statistical model cannot evaluate the relative importance of different interrelated nodes in the network. The existing researches regards hypoglycemic fear as a whole and studies the relationship between it and other variables [26, 27] which will lead to spurious correlations among variables easily appear when there are more variables. So, we do not know how the items in the two dimensions of hypoglycemic fear, hypoglycemic worries and hypoglycemic avoidance, interact with each other.

Network analysis can reduce these problems by fitting data with Gaussian image model [28]. It is based on mathematical analysis and visual representation of the interaction between complex variables. The advantage of network analysis is that it does not rely on previous assumptions about the relationship between variables, and allows visualization of the association patterns of different variables [29]. Community is always used to represent a group of psychological variables. The bridge centrality index helps to accurately capture variables that play a key role in connecting communities in the whole network [30, 31].

Therefore, we will rely on a network analysis approach to analyze the relationship between hypoglycemia worries and avoiding hypoglycemia behavior in T2DM patients with hypoglycemia. We constructed the network model and estimated the bridge centrality to detect the important role of some specific aspects of the effect of hypoglycemic worries on avoiding hypoglycemic behavior and identified the variables connecting these two communities to reduce hypoglycemic worries and avoid hypoglycemic behavior excessively in T2DM patients with hypoglycemia.

## Materials and methods

### Participants

A total of 300 T2DM patients with hypoglycemia from 2 hospitals participated in a cross-sectional survey between October 2022 and December 2022; the T2DM patients were recruited via the convenience sampling method. Patients who met the following inclusion criteria were included in the research: (1) were diagnosed with type 2 diabetes; (2) without hearing or reading impairment or without cognitive dysfunction (since the research data was collected by answering questionnaires); (3) suffered from hypoglycemia within half a year (in order to avoid memory bias); (4) gave informed consent and volunteered to participate in the research. The exclusive criteria were patients with type 2 diabetes who were not using drugs with high hypoglycemic risks, such as insulin or sulfonylureas. The general information of T2DM patients is shown in Table 1.

**Table 1:** Demographic characteristics of T2DM patients with hypoglycemia (N = 283)

Variables		N	Percentage (%)
**Sex**	Male	154	54.42
	Female	129	45.58
**Age**	18 ~ 20	4	1.41
	20 ~ 30	15	5.30
	31 ~ 40	68	24.03
	> 40	196	69.26
**Educational level**	Primary school or below	89	31.45
	Junior or Senior high school	125	44.17
	Bachelor or above	69	24.38
**Marital status**	Unmarried	31	10.95
	Married	206	72.79
	Divorced/widowed	46	16.25
**Wages (Yuan a month)**	< 3000	68	24.03
	3000 ~ 5000	134	47.35
	> 5000	81	28.62
**Place of residence**	Urban area	126	44.52
	Rural area	157	55.48
**Live alone**	Yes	79	27.92
	No	204	72.08
**Duration of diabetes**	< 3	75	26.50
	3 ~ 5	69	24.38
	6 ~ 10	89	31.45
	> 10	50	17.67
**Frequency of hypoglycemia**	1 ~ 2 times/6 months	93	32.86
	3 ~ 6 times/6 months	124	43.82
	More than 6 time/6 months	66	23.32

According to the calculation, the sample size was at least 5 times of the number of items in the questionnaire. There were 33 variables in the study. Therefore, the calculation formula of sample size was N = 33*5 = 165, which meant that at least 165 subjects were required for this study.

### Data collection

Before the investigation, purpose of the study was explained to the participants and verbal consent was retrieved from the participants. Besides, the participants also signed the informed consent form. In the process of the investigation, participants could terminate and withdraw from the investigation at any time and the questionnaire was completed anonymously. They were assured that their information would only be used for research. According to the voluntary principle, 300 T2DM patients with hypoglycemia participated in the survey. The digital version of the questionnaire was issued to the participants. Among the 300 T2DM patients, 13 dropped out of the research, and 4 did not complete the questionnaire. Eventually, 283 questionnaires were valid, with an effective response rate of 94.33%.

### Measurements

The Chinese version of the Hypoglycemia Fear Survey II (HFS-II) was developed by Cox [32]. It was translated into Chinese and was widely used in China [33, 34]. It is a 5-point Likert scale consisting of an 18-item Chinese version of the Hypoglycemia Fear Survey II- hypoglycemia worries scale (CHFSII-WS) and a 15-item avoiding hypoglycemia behavior scale (CHFSII-BS). The worries scale reflects fears or worries about hypoglycemia of patients with diabetes, and the behavior subscale contains behaviors that patients use to avoid hypoglycemia. The scores of the HFS-II, CHFSII-WS and CHFSII-BS ranged from 0 ~ 132, 0 ~ 72 and 0 ~ 60, respectively. High scores indicate a high level of hypoglycemia fear. The Cronbach’s alphas coefficient of the HFS-II was 0.876, while that of each dimension was 0.893 and 0.861, respectively.

### Data analysis

We adopted SPSS 26.0 and R 4.1.1 to analysis the collected data. SPSS 26.0 software was used to analyze sociodemographic characteristics, hypoglycemia worries, avoiding hypoglycemia behavior and other descriptive data. R 4.1.1 software was used to construct the network model and estimate the bridge centrality to identify the bridge items between hypoglycemia worries and avoiding hypoglycemia behavior.

#### Network model

The network of hypoglycemia worries and avoiding hypoglycemia behavior was constructed by the R package qgraph [35]. Least Absolute Shrinkage and Selection Operator (LASSO) [36] regularization and the Extended Bayesian Information Criterion (EBIC) [37] were used to shrink minor edges to zero weight [38]. Spearman’s correlation was adopted, and the EBIC hyperparameter was set to 0.500. In the network, nodes represent different contents of hypoglycemia worries and avoiding hypoglycemia behavior. Lines represent statistical relationships between these contents, the influence on the correlation of any two nodes caused by other nodes was eliminated by statistical control [39]. According to the subordination of items represented by the nodes, the nodes were grouped into different communities [40]. The hypoglycemia worries community consisted of 18 nodes, while the avoiding hypoglycemia behavior community consisted of 15 nodes. The accuracy test and the difference significance test of edge weight indices were conducted by the R package *bootnet* [41].

#### Bridge centrality

The bridge centrality was estimated by R package networktools [42]. The bridge expected influence (BEI) of a node is defined as the sum of the connections between the node and all nodes from another community [43]. The stability test and difference significance test of the BEI indices were conducted by the R package *bootnet*, and the stability was quantitatively evaluated by the correlation stability coefficient (CS-coefficient). The value of correlation stability coefficient preferably should be above 0.5 and should not be below 0.25 [44].

## Results

### Descriptive Statistics

Among the 283 T2DM patients with hypoglycemia, the mean (SD) age was 47.64 ± 11.24 years. 54.42% were male, and 45.58% were female. 31.45% had a primary school education or below, 44.17% had a junior or senior high school education, and 24.38% had a bachelor education or above. 26.50% patients had diabetes for less than 3 years, 24.38% had diabetes for 3 ~ 5 years, 31.45% had diabetes for 6 ~ 10 years, 17.67% had diabetes for more than 10 years. 32.86% had hypoglycemia once or twice in half a year; 43.82% had hypoglycemia three to six times in half a year; 23.32% had hypoglycemia more than six times in half a year. Other general demographic data are shown in Table 1. Moreover, the mean scores, standard deviations and abbreviations for each item for hypoglycemia worries and avoiding hypoglycemia behavior are presented in Table 2.

**Table 2:** Mean scores, standard deviations and abbreviations for each item on hypoglycemia worries and avoiding hypoglycemia behavior

Items	Abbreviation	M	SD	BEI
**Hypoglycemia worries**				
I am afraid that I will have hypoglycemia, but I do not realize it.	W1	2.62	1.05	0.11
I am worried that there is no food, fruit, or drink within my reach.	W2	2.65	1.15	0.00
I am afraid I’ll faint in public.	W3	1.47	0.80	0.13
I am worried that hypoglycemia will embarrass me or my friends in public.	W4	1.55	0.88	0.02
I am worried about hypoglycemia when I’m alone.	W5	2.50	0.88	0.00
I am afraid I will be in a trance when hypoglycemia occurs.	W6	1.71	0.89	0.04
I am afraid I lose control after hypoglycemia.	W7	1.77	0.94	0.06
I am afraid that when I have hypoglycemia, there is no one around to help me.	W8	1.85	0.98	0.05
I am worried about hypoglycemia when I’m driving or cycling.	W9	1.50	0.83	0.01
I am afraid of causing mistakes or accidents due to hypoglycemia.	W10	1.40	0.73	0.08
I am worried about being criticized by others because of hypoglycemia.	W11	1.51	0.82	0.00
I am worried that hypoglycemia will affect my judgment.	W12	2.46	1.31	0.21
I am afraid hypoglycemia makes me feel dizzy.	W13	2.69	1.08	0.07
I am worried that hypoglycemia may hurt me or others.	W14	1.38	0.77	0.16
I am worried that hypoglycemia will cause permanent damage to my body or health.	W15	1.44	0.87	0.09
I am worried that the occurrence of hypoglycemia will disrupt some important things I am doing.	W16	2.62	1.32	0.09
I worry about hypoglycemia during sleep.	W17	1.72	0.92	0.39
I am worried that hypoglycemia will make me suddenly feel restless and difficult to calm down.	W18	2.37	0.67	0.00
**Avoiding hypoglycemia behavior**				
When I feel signs of hypoglycemia, I will eat something.	B1	3.47	1.11	-0.08
I will keep my fasting blood sugar above 8 mmol/L to avoid hypoglycemia.	B2	2.27	1.23	0.07
When my blood sugar is low, I will reduce the dose of insulin or drugs.	B3	2.51	1.28	0.00
I will increase the frequency of blood glucose monitoring.	B4	2.92	1.16	0.00
Worried about hypoglycemia, I will ensure that I am accompanied when I go out.	B5	2.14	1.06	0.20
Worried about hypoglycemia, I will reduce my outing or travel.	B6	2.43	1.17	0.11
Worried about hypoglycemia, I dare not drive a car or bicycle.	B7	2.26	1.40	0.11
Worried about hypoglycemia, I’m afraid to visit relatives and friends.	B8	2.11	1.17	0.04
Had to stay at home for fear of hypoglycemia.	B9	1.99	1.19	0.35
Restricted exercise and physical activity due to fear of hypoglycemia.	B10	2.15	1.20	0.26
Make sure there are people around who can help me.	B11	1.96	1.12	0.05
Take candy or carbohydrates to avoid hypoglycemia.	B12	3.11	1.36	0.00
Keep blood sugar higher than usual when participating in some activities.	B13	2.10	1.12	0.01
When doing important work, keep the blood sugar higher than usual.	B14	2.12	1.09	0.10
Seek the attention of others.	B15	1.75	1.01	0.27

### Network structure

#### The characteristics of edges

**Fig. 1:**
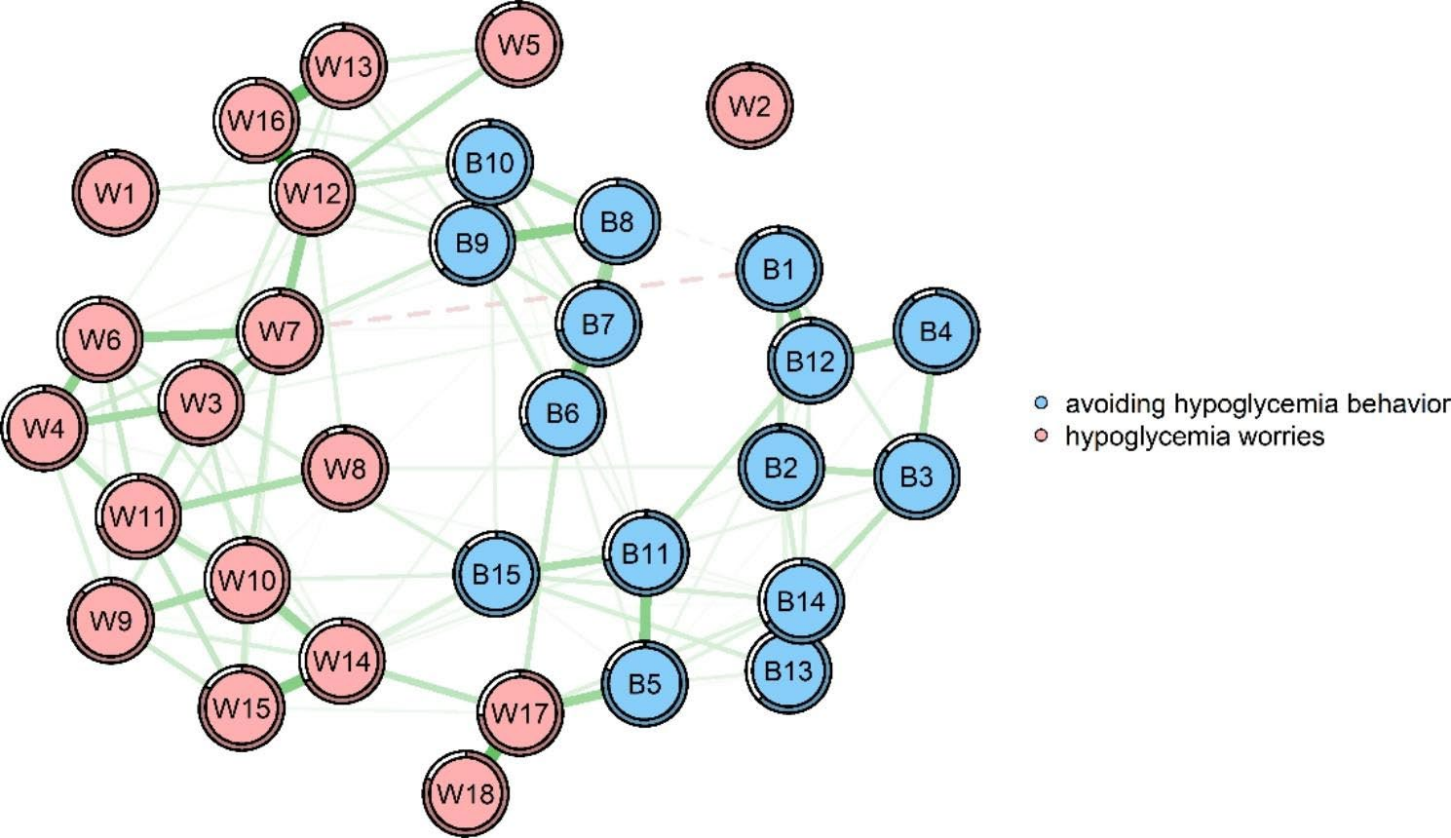
Network structure of hypoglycemia worries and avoiding hypoglycemia behavior. Green edges represent positive correlations, red edges represent negative correlations. The thickness of the edge reflects the magnitude of the correlation. The circles around nodes depict its predictability. W1: I am afraid that I will have hypoglycemia, but I do not realize it; W2: I am worried that there is no food, fruit, or drink within my reach; W3: I am afraid I’ll faint in public; W4: I am worried that hypoglycemia will embarrass me or my friends in public; W5: I am worried about hypoglycemia when I’m alone; W6: I am afraid I will be in a trance when hypoglycemia occurs; W7: I am afraid I lose control after hypoglycemia; W8: I am afraid that when I have hypoglycemia, there is no one around to help me; W9: I am worried about hypoglycemia when I’m driving or cycling; W10: I am afraid of causing mistakes or accidents due to hypoglycemia; W11: I am worried about being criticized by others because of hypoglycemia; W12: I am worried that hypoglycemia will affect my judgment; W13: I am afraid hypoglycemia makes me feel dizzy; W14: I am worried that hypoglycemia may hurt me or others; W15: I am worried that hypoglycemia will cause permanent damage to my health; W16: I am worried that the occurrence of hypoglycemia will disrupt some important things I am doing; W17: I worry about hypoglycemia during sleep; W18: I am worried that hypoglycemia will make me suddenly feel restless and difficult to calm down; B1: When I feel signs of hypoglycemia, I will eat something; B2: I will keep my fasting blood sugar above 8 mmol/L to avoid hypoglycemia; B3: When my blood sugar is low, I will reduce the dose of insulin or drugs; B4: I will increase the frequency of blood glucose monitoring; B5: Worried about hypoglycemia, I will ensure that I am accompanied when I go out; B6: Worried about hypoglycemia, I will reduce my outing or travel; B7: Worried about hypoglycemia, I dare not drive a car or bicycle; B8: Worried about hypoglycemia, I’m afraid to visit relatives and friends; B9: Had to stay at home for fear of hypoglycemia; B10: Restricted exercise and physical activity due to fear of hypoglycemia; B11: Make sure there are people around who can help me; B12: Take candy or carbohydrates to avoid hypoglycemia.; B13: Keep blood sugar higher than usual when participating in some activities; B14: When doing important work, keep the blood sugar higher than usual; B15: Seek the attention of others

The network model is shown in Fig. 1. There was total 129 edges (range from − 0.08 to 0.43) in the whole network and 37 edges (range from − 0.08 to 0.20) across the hypoglycemia worries and avoiding hypoglycemia behavior communities in the network.

In the edges across communities, W1 was positively correlated with 2 nodes of the avoiding hypoglycemia behavior community, namely, B9 and B10, among which the correlation with B10 was stronger (edge weight: 0.07). W3 was positively correlated with 3 nodes of the avoiding hypoglycemia behavior community, namely, B7, B9, and B15, among which the correlation with B15 was the strongest (edge weight: 0.08). W4 was positively correlated with 1 node of the avoiding hypoglycemia behavior community, namely, B14 (edge weight: 0.02). W6 was positively correlated with 3 nodes of the avoiding hypoglycemia behavior community, namely, B7, B8, and B10, among which the correlation with B10 was the strongest (edge weight: 0.03). W7 was positively correlated with 3 nodes of the avoiding hypoglycemia behavior community, namely, B7-B9, among which the correlation with B9 was the strongest (edge weight: 0.10), and was negatively correlated with B1 of the avoiding hypoglycemia behavior community (edge weight: -0.08). W8 was positively correlated with 1 node of the avoiding hypoglycemia behavior community, namely, B2 (edge weight: 0.05). W9 was positively correlated with 1 node of the avoiding hypoglycemia behavior community, namely, B14 (edge weight: 0.01). W10 was positively correlated with 3 nodes of the avoiding hypoglycemia behavior community, namely, B8, B9, and B15, among which the correlation with B15 was the strongest (edge weight: 0.07). W12 was positively correlated with 2 nodes of the avoiding hypoglycemia behavior community, namely, B9 and B10, among which the correlation with B10 was stronger (edge weight: 0.11). W13 was positively correlated with 2 nodes of the avoiding hypoglycemia behavior community, namely, B7 and B9, among which the correlation with B9 was stronger (edge weight: 0.06). W14 was positively correlated with 4 nodes of the avoiding hypoglycemia behavior community, namely, B2, B7, B11 and B15, among which the correlation with B15 was the strongest (edge weight: 0.06). W15 was positively correlated with 5 nodes of the avoiding hypoglycemia behavior community, namely, B6, B9, B11, B13 and B15, among which the correlation with B15 was the strongest (edge weight: 0.07). W16 was positively correlated with 2 nodes of the avoiding hypoglycemia behavior community, namely, B9 and B10, among which the correlation with B10 was stronger (edge weight: 0.06). W17 was positively correlated with 4 nodes of the avoiding hypoglycemia behavior community, namely, B5, B6, B13 and B14, among which the correlation with B5 was the strongest (edge weight: 0.20). See Supplementary Table 1 of the supplemental material for more detailed information on the correlations among nodes in the network. For edges in hypoglycemia worries community, there were 92 edges ranging from < 0.01 to 0.43, and the strongest correlation was between W12 and W16. For edges in the avoiding hypoglycemia behavior community, there were 90 edges ranging from − 0.03 to 0.52, and the strongest correlation was between B13 and B14.

In the network of hypoglycemia worries and avoiding hypoglycemia behavior, the 95% confidence interval of edge weights was narrow, which indicated that the accuracy of edge weights was acceptable (see Supplementary Fig. 1 in supplementary material). As the result of the difference significance test of the edge weight showed, the edge weight between W17 and B5 was the largest among the cross-community edges (see Supplementary Fig. 2 in supplementary material).

#### The characteristics of nodes

**Fig. 2:**
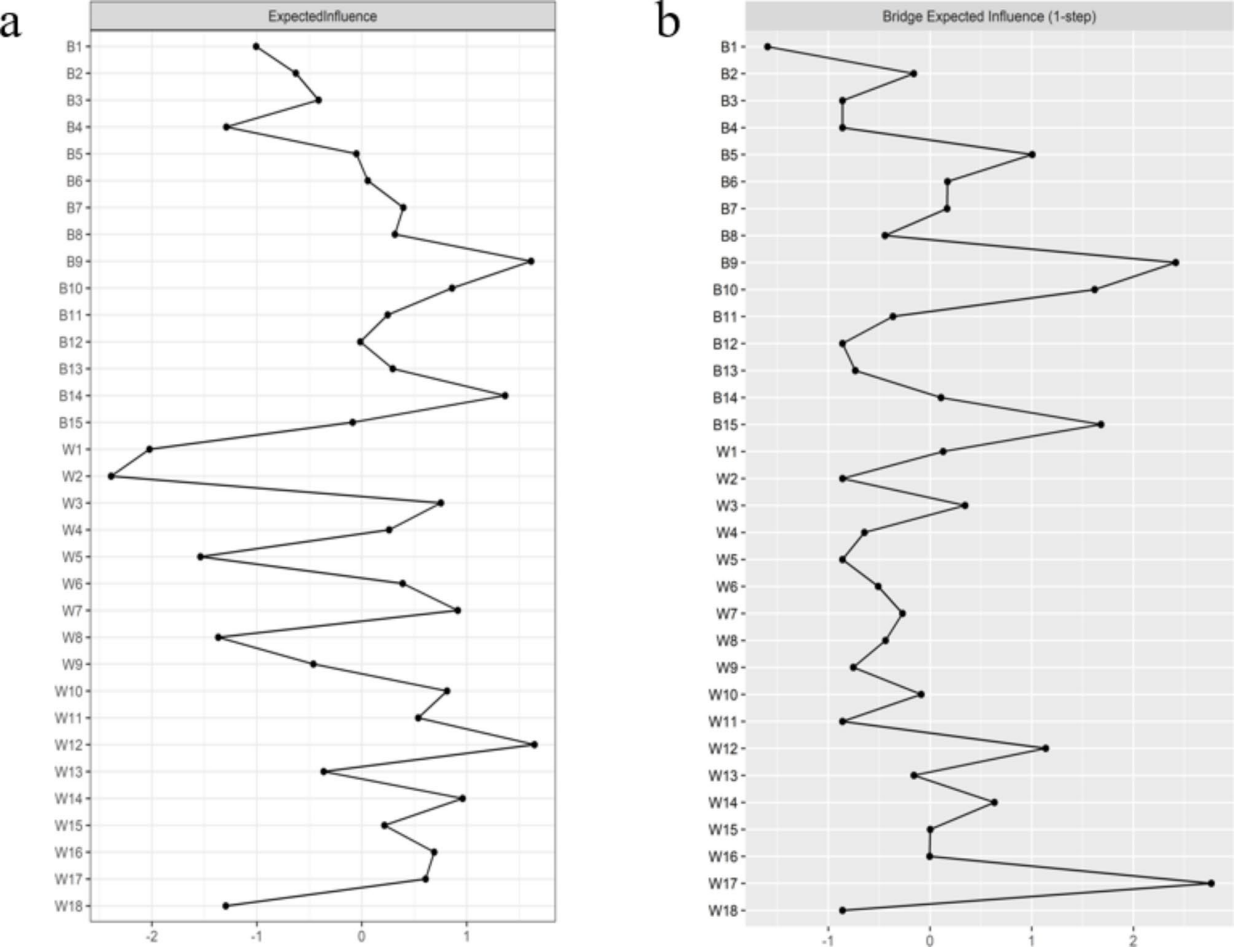
Centrality plot depicting the expected influence and bridge expected influence of each variable selected in the present network (z-score). W1: I am afraid that I will have hypoglycemia, but I do not realize it; W2: I am worried that there is no food, fruit, or drink within my reach; W3: I am afraid I’ll faint in public; W3: I am afraid I’ll faint in public; W4: I am worried that hypoglycemia will embarrass me or my friends in public; W5: I am worried about hypoglycemia when I’m alone; W6: I am afraid I will be in a trance when hypoglycemia occurs;; W7: I am afraid I lose control after hypoglycemia; W8: I am afraid that when I have hypoglycemia, there is no one around to help me; W9: I am worried about hypoglycemia when I’m driving or cycling; W10: I am afraid of causing mistakes or accidents due to hypoglycemia; W11: I am worried about being criticized by others because of hypoglycemia; W12: I am worried that hypoglycemia will affect my judgment; W13: I am afraid hypoglycemia makes me feel dizzy; W14: I am worried that hypoglycemia may hurt me or others; W15: I am worried that hypoglycemia will cause permanent damage to my health; W16: I am worried that the occurrence of hypoglycemia will disrupt some important things I am doing; W17: I worry about hypoglycemia during sleep; W18: I am worried that hypoglycemia will make me suddenly feel restless and difficult to calm down; B1: When I feel signs of hypoglycemia, I will eat something; B2: I will keep my fasting blood sugar above 8 mmol/L to avoid hypoglycemia; B3: When my blood sugar is low, I will reduce the dose of insulin or drugs; B4: I will increase the frequency of blood glucose monitoring; B5: Worried about hypoglycemia, I will ensure that I am accompanied when I go out; B6: Worried about hypoglycemia, I will reduce my outing or travel; B7: Worried about hypoglycemia, I dare not drive a car or bicycle; B8: Worried about hypoglycemia, I’m afraid to visit relatives and friends; B9: Had to stay at home for fear of hypoglycemia; B10: Restricted exercise and physical activity due to fear of hypoglycemia; B11: Make sure there are people around who can help me; B12: Take candy or carbohydrates to avoid hypoglycemia.; B13: Keep blood sugar higher than usual when participating in some activities; B14: When doing important work, keep the blood sugar higher than usual; B15: Seek the attention of others

The node expected influence is shown in Fig. 2(a). W12 “I am worried that hypoglycemia will affect my judgment” and B9 “Had to stay at home for fear of hypoglycemia” have the highest expected influence, indicating that these two variables are the most associated nodes in the present network from the perspective of statistics. W2 “I am worried that there is no food, fruit, or drink within my reach” of hypoglycemia worries has the lowest expected influence, indicating that this variable is the least associated node in the present network from the perspective of statistics. As shown in Supplementary Fig. 3 of the supplementary material, with the reduction of the subsample, the average correlation of the BEI indices of the original sample and the subsample decreased, while the CS coefficient was 0.284, which was larger than 0.25 and indicated acceptable stability. As shown in Supplementary Fig. 4 of the supplementary material, the BEIs of W17 “I worry about hypoglycemia during sleep” and B9 “Had to stay at home for fear of hypoglycemia” were statistically larger than those of at least 74% of the other nodes (*P* < 0.05).

The node bridge expected influence is shown in Fig. 2(b). In the community of hypoglycemia worries, W17 “I worry about hypoglycemia during sleep” has the highest bridge expected influence, which indicates it has the strongest connections with avoiding hypoglycemia behavior. In the community of avoiding hypoglycemia behavior, B9 “Had to stay at home for fear of hypoglycemia” has the highest bridge expected influence, which indicates it has the strongest connections with hypoglycemia worries.

## Discussion

Employing network analysis, we aim to reveal potential pathways that how different components of hypoglycemia worries are related to the avoiding hypoglycemia behavior. It is observed that different items of hypoglycemia worries are commonly but differentially related to items of avoiding hypoglycemia behavior. The results suggest that different items of hypoglycemia worries may have similar and specific pathways leading to avoiding hypoglycemia behavior. In some extent, our finding adds to emerging research showing that how hypoglycemic worries affects hypoglycemic avoidance behavior and how they interact.

Within components of hypoglycemia worries, the present study finds a strongest exists between W12 “I am worried that hypoglycemia will affect my judgment” and W16 “I am worried that the occurrence of hypoglycemia will disrupt some important things I am doing”. These two items are the influence of hypoglycemia on individual cognitive ability and behavior. This is consistent with the results of Lara Coelho’s research, which shows that individuals’ cognitive impairment will limit their actions [45]. The other three strongest edges are between W17 “I worry about hypoglycemia during sleep” and W18 “I am worried that hypoglycemia will make me suddenly feel restless and difficult to calm down”, between W13 “I am afraid hypoglycemia makes me feel dizzy” and W16 “I am worried that the occurrence of hypoglycemia will disrupt some important things I am doing”, and between W14 “I am worried that hypoglycemia may hurt me or others” and W15 “I am worried that hypoglycemia will cause permanent damage to my health”. These worries items reflect the harm of hypoglycemia to individual health and behavior. Within items of hypoglycemic avoidance behavior, the present study finds a strongest exists between B13 “Keep blood sugar higher than usual when participating in some activities” and B14 “When doing important work, keep the blood sugar higher than usual”. Both of these two items are about individuals keeping their blood sugar at a high level for fear of hypoglycemia, which is consistent with the results of previous study on behavior of hypoglycemic patients [46].

W12 “I am worried that hypoglycemia will affect my judgment” of hypoglycemia worries and B9 “Had to stay at home for fear of hypoglycemia” of avoiding hypoglycemia behavior have the highest expected influences which indicates these two variables may play the most important role in the present hypoglycemia fear network consisting 18 items of hypoglycemia worries and 15 items of avoiding hypoglycemia behavior. These two variables indicate that they may be a good breakthrough point to reduce T2DM patients’ fear of hypoglycemia. The occurrence of hypoglycemia in diabetes patients will bring many serious problems, such as the damage to cognitive function and judgment [47, 48]. This also causes patients to fear the harm caused by hypoglycemia and dare not go out [49, 50]. T2DM patients with hypoglycemia are a special and large group, lacking a sense of security, so they are afraid of hypoglycemia and often take excessive measures to avoid hypoglycemia [51].

In the current network, node bridge strength centrality may cast light on the specific role played by these different items of hypoglycemia worries in the context of avoiding hypoglycemia behavior. In the community of hypoglycemia worries, W17 “I worry about hypoglycemia during sleep” has the highest bridge expected influence. This suggests that W17 “I worry about hypoglycemia during sleep” has stronger associations with avoiding hypoglycemia behavior community than other items. Thus, from a network perspective, targeting W17 “I worry about hypoglycemia during sleep” may be more effective at reducing excessive avoiding hypoglycemia behavior than targeting other items of hypoglycemia worries. Therefore, clinical medical staff should help diabetes patients to keep their blood sugar stable through scientific medication and correct monitoring, and educate patients to eat dinner to prevent nocturnal hypoglycemia [52, 53]. In the community of avoiding hypoglycemia behavior, B9 “Had to stay at home for fear of hypoglycemia” has the highest bridge expected influence. This suggests that B9 “Had to stay at home for fear of hypoglycemia” has stronger associations with hypoglycemia worries community than other items. It is wrong to limit activities to stay at home for fear of hypoglycemia. Research has confirmed that moderate exercise can help diabetes patients better control blood sugar [54, 55]. Therefore, medical staff should encourage diabetes patients to take an active part in exercise, and should not stay at home for fear of hypoglycemia [56]. Help patients overcome their hypoglycemic concerns by encouraging them to go out of the room.

The present study provides a fine-grained understanding of the relations between hypoglycemic worries and excessive hypoglycemic avoidance behavior. We broke through the existing research to take hypoglycemic fear as a whole and explore the relationship between hypoglycemic worries and excessive hypoglycemic avoidance behavior from its internal perspective. All T2DM patients should be encouraged to discuss their experiences with hypoglycemia without judgment or shame. Glucose targets, testing schedules and treatment plans should be reviewed often and individualized to the minimize risk of hypoglycemia. Medical staff should eliminate diabetes patients’ hypoglycemia worries, reduce excessive hypoglycemic avoidance behaviors, and help patients improve their quality of life.

## Limitations

There are some limitations to our study. Firstly, our study is conducted in the form of self-report questionnaire, and the results are relatively subjective. Secondly, our study chose the convenient sampling method for the reason that there are too many hospitals in China and the research fund of our study is limited. In future studies, we will choose stratified random sampling method, which may provide a more scientific result, especially in the massive investigation. Lastly, the network in this study estimated between-subject effects on a group level. Thus, it is possible that characteristics, such as centrality and network structure, may not remain the same on an individual level.

## Conclusion

In summary, our study provides an illustration of the richness and complexity of the associations involved in the structure of hypoglycemia worries and excessive avoiding hypoglycemia behavior in T2DM patients with hypoglycemia. In the network, different aspects of hypoglycemia worries were correlated with different aspects of excessive avoiding hypoglycemia behavior. From the perspective of network analysis, B9 “Had to stay at home for fear of hypoglycemia” and W12 “I am worried that hypoglycemia will affect my judgment” have the highest expected influence, indicating their highest importance in the network. W17 “I worry about hypoglycemia during sleep” aspect of hypoglycemia worries and B9 “Had to stay at home for fear of hypoglycemia” aspect of avoiding hypoglycemia behavior have the highest bridge expected influence, indicating they have the strongest connections with each community. These results have important implications to clinical practice, which provided potential targets for interventions to reduce hypoglycemia fear and improve the quality of life in T2DM patients with hypoglycemia.

## Electronic supplementary material

Below is the link to the electronic supplementary material.

Supplementary Material 1

## Data Availability

The datasets used and analyzed during the current study are available from thr corresponding author on reasonable request.

## Abbreviations

W1: I am afraid that I will have hypoglycemia, but I do not realize it
W2: I am worried that there is no food, fruit, or drink within my reach
W3: I am afraid I’ll faint in public
W3: I am afraid I’ll faint in public
W4: I am worried that hypoglycemia will embarrass me or my friends in public
W5: I am worried about hypoglycemia when I’m alone
W6: I am afraid I will be in a trance when hypoglycemia occurs
W7: I am afraid I lose control after hypoglycemia
W8: I am afraid that when I have hypoglycemia, there is no one around to help me
W9: I am worried about hypoglycemia when I’m driving or cycling
W10: I am afraid of causing mistakes or accidents due to hypoglycemia
W11: I am worried about being criticized by others because of hypoglycemia
W12: I am worried that hypoglycemia will affect my judgment
W13: I am afraid hypoglycemia makes me feel dizzy
W14: I am worried that hypoglycemia may hurt me or others
W15: I am worried that hypoglycemia will cause permanent damage to my health
W16: I am worried that the occurrence of hypoglycemia will disrupt some important things I am doing
W17: I worry about hypoglycemia during sleep
W18: I am worried that hypoglycemia will make me suddenly feel restless and difficult to calm down
B1: When I feel signs of hypoglycemia, I will eat something
B2: I will keep my fasting blood sugar above 8 mmol/L to avoid hypoglycemia
B3: When my blood sugar is low, I will reduce the dose of insulin or drugs
B4: I will increase the frequency of blood glucose monitoring
B5: Worried about hypoglycemia, I will ensure that I am accompanied when I go out
B6: Worried about hypoglycemia, I will reduce my outing or travel
B7: Worried about hypoglycemia, I dare not drive a car or bicycle
B8: Worried about hypoglycemia, I’m afraid to visit relatives and friends
B9: Had to stay at home for fear of hypoglycemia
B10: Restricted exercise and physical activity due to fear of hypoglycemia
B11: Make sure there are people around who can help me
B12: Take candy or carbohydrates to avoid hypoglycemia
B13: Keep blood sugar higher than usual when participating in some activities
B14: When doing important work, keep the blood sugar higher than usual
B15: Seek the attention of others

## References

[1] Ali MK, Pearson-Stuttard J, Selvin E, Gregg EW (2022). Interpreting global trends in type 2 diabetes complications and mortality. Diabetologia.

[2] Rizzo MR, Di Meo I, Polito R, Auriemma MC, Gambardella A, di Mauro G, Capuano A, Paolisso G (2022). Cognitive impairment and type 2 diabetes mellitus: focus of SGLT2 inhibitors treatment. Pharmacol Res.

[3] Yu J, Yi Q, Chen G, Hou L, Liu Q, Xu Y, Qiu Y, Song P (2022). The visceral adiposity index and risk of type 2 diabetes mellitus in China: a national cohort analysis. Diab/Metab Res Rev.

[4] Kanaley JA, Colberg SR, Corcoran MH, Malin SK, Rodriguez NR, Crespo CJ, Kirwan JP, Zierath JR (2022). Exercise/Physical activity in individuals with type 2 diabetes: a Consensus Statement from the American College of Sports Medicine. Med Sci Sports Exerc.

[5] Han H, Lai J, Yan C, Li X, Hu S, He Y, Li H (2022). Development and validation of a prediction model of perioperative hypoglycemia risk in patients with type 2 diabetes undergoing elective surgery. BMC Surg.

[6] Wu C, Ge YL, Zhang XY, Liu MC, Heng CN, Zhang LY, Du YL, He SZ, Shang L, Lang HJ (2021). The influence of hypoglycemia on the specific quality of life in type 2 diabetes mellitus: a comparative cross-sectional study of diabetics with and without hypoglycemia in Xi’an, China. Health Qual Life Outcomes.

[7] Gardner CD, Landry MJ, Perelman D, Petlura C, Durand LR, Aronica L, Crimarco A, Cunanan KM, Chang A, Dant CC (2022). Effect of a ketogenic diet versus Mediterranean diet on glycated hemoglobin in individuals with prediabetes and type 2 diabetes mellitus: the interventional Keto-Med randomized crossover trial. Am J Clin Nutr.

[8] Wang F, Mao Y, Wang H, Liu Y, Huang P (2022). Semaglutide and Diabetic Retinopathy Risk in patients with type 2 diabetes Mellitus: a Meta-analysis of Randomized controlled trials. Clin Drug Investig.

[9] Steinbrenner H, Duntas LH, Rayman MP (2022). The role of selenium in type-2 diabetes mellitus and its metabolic comorbidities. Redox Biol.

[10] Echouffo-Tcheugui JB, Kaze AD, Fonarow GC, Dagogo-Jack S (2022). Severe hypoglycemia and Incident Heart failure among adults with type 2 diabetes. J Clin Endocrinol Metab.

[11] Andersen A, Bagger JI, Sørensen SK, Baldassarre MPA, Pedersen-Bjergaard U, Forman JL, Gislason G, Lindhardt TB, Knop FK, Vilsbøll T (2021). Associations of hypoglycemia, glycemic variability and risk of cardiac arrhythmias in insulin-treated patients with type 2 diabetes: a prospective, observational study. Cardiovasc Diabetol.

[12] Yun JS, Han K, Ko SH (2022). Trends of severe hypoglycemia in patients with type 2 diabetes in Korea: a longitudinal nationwide cohort study. J diabetes Invest.

[13] Lambert-Obry V, Lafrance JP, Savoie M, Lachaine J (2022). The impact of Hypoglycemia on Productivity loss and utility in patients with type 2 diabetes treated with insulin in real-world canadian practice: protocol for a prospective study. JMIR Res protocols.

[14] Heller SR, Pratley RE, Sinclair A, Festa A, Kiljański J, Brusko CS, Duan R, Heine RJ (2018). Glycaemic outcomes of an individualized treatMent aPproach for oldER vulnerable patIents: a randomized, controlled stUdy in type 2 diabetes Mellitus (IMPERIUM). Diabetes Obes Metab.

[15] Siamashvili M, Davis S (2021). Late phase completed clinical trials investigating bromocriptine mesylate quick release as treatment of type 2 diabetes mellitus. Expert Opin Pharmacother.

[16] Zhang Y, Li S, Zou Y, Wu X, Bi Y, Zhang L, Yuan Y, Gong W, Hayter M. Fear of hypoglycemia in patients with type 1 and 2 diabetes: a systematic review.Journal of clinical nursing2020. 10.1111/jocn.15538 33091198

[17] Muneer M. Hypoglycaemia. *Advances in experimental medicine and biology* 2021, 1307:43–69. 10.1007/5584_2020_534 32406022

[18] Polonsky WH, Fisher L, Hessler D, Edelman SV (2015). Identifying the worries and concerns about hypoglycemia in adults with type 2 diabetes. J Diabetes Complicat.

[19] Shi Min Ko M, Kit Lee W, Chang Ang L, Goh SY, Mong Bee Y, Ming Teh M (2022). A cross-sectional study on risk factors for severe hypoglycemia among insulin-treated elderly type 2 diabetes Mellitus (T2DM) patients in Singapore. Diabetes Res Clin Pract.

[20] Sheu WH, Ji LN, Nitiyanant W, Baik SH, Yin D, Mavros P, Chan SP (2012). Hypoglycemia is associated with increased worry and lower quality of life among patients with type 2 diabetes treated with oral antihyperglycemic agents in the Asia-Pacific region. Diabetes Res Clin Pract.

[21] Grammes J, Schäfer M, Benecke A, Löw U, Klostermann AL, Kubiak T, Witthöft M (2018). Fear of hypoglycemia in patients with type 2 diabetes: the role of interoceptive accuracy and prior episodes of hypoglycemia. J Psychosom Res.

[22] O’Donnell HK, Bennett Johnson S, Sileo D, Majidi S, Gonder-Frederick L, Driscoll KA (2022). Psychometric Properties of the Hypoglycemia Fear Survey in a clinical sample of adolescents with type 1 diabetes and their caregivers. J Pediatr Psychol.

[23] Polonsky WH, Fisher L, Hessler D, Liu J, Fan L, McAuliffe-Fogarty AH (2020). Worries and concerns about hypoglycemia in adults with type 1 diabetes: an examination of the reliability and validity of the hypoglycemic attitudes and Behavior Scale (HABS). J Diabetes Complicat.

[24] Roberts AJ, Yi-Frazier JP, Carlin K, Taplin CE (2020). Hypoglycaemia avoidance behaviour and exercise levels in active youth with type 1 diabetes. Endocrinol diabetes metabolism.

[25] Wieringa TH, de Wit M, Twisk JWR, Snoek FJ (2018). Does hypoglycaemia affect the improvement in QoL after the transition to insulin in people with type 2 diabetes?. J Endocrinol Investig.

[26] Przezak A, Bielka W, Molęda P (2022). Fear of hypoglycemia-An underestimated problem. Brain and behavior.

[27] Nakhleh A, Shehadeh N (2021). Hypoglycemia in diabetes: an update on pathophysiology, treatment, and prevention. World J diabetes.

[28] Serra B, Mendoza M, Scazzocchio E, Meler E, Nolla M, Sabrià E, Rodríguez I, Carreras E. A new model for screening for early-onset preeclampsia. *American journal of obstetrics and gynecology* 2020, 222(6):608.e601-608.e618. 10.1016/j.ajog.2020.01.020 31972161

[29] Askar M, Cañadas RN, Svendsen K (2021). An introduction to network analysis for studies of medication use. Res social administrative pharmacy: RSAP.

[30] Vanzhula IA, Kinkel-Ram SS, Levinson CA (2021). Perfectionism and Difficulty Controlling Thoughts Bridge eating disorder and obsessive-compulsive disorder symptoms: A Network Analysis. J Affect Disord.

[31] Stevens NA, Lydon M, Marshall AH, Taylor S. Identification of Bridge Key Performance Indicators Using Survival Analysis for Future Network-Wide Structural Health Monitoring.Sensors (Basel, Switzerland)2020, 20(23). 10.3390/s20236894 PMC7731222 33276606

[32] Cox DJ, Irvine A, Gonder-Frederick L, Nowacek G, Butterfield J (1987). Fear of hypoglycemia: quantification, validation, and utilization. Diabetes Care.

[33] Li S, Fang L, Lee A, Hayter M, Zhang L, Bi Y, Wu X, Liu L, Zhang H, Yuan Y (2021). The association between diabetes-related distress and fear of hypoglycaemia in patients with type 2 diabetes mellitus: a cross-sectional descriptive study. Nurs open.

[34] Huang J, Peng W, Ding S, Xiong S, Liu Z (2022). Fear of hypoglycemia and associated factors in hospitalized patients with type 2 diabetes: a cross–sectional study. Sci Rep.

[35] Epskam S, Cramer AOJ, Waldorp LJ, Schmittmann VD, Borsboom D. Qgraph: network visualizations of relationships in psychometric data. J Stat Softw. 2012;48(4):1–18.

[36] Ma X, Mo C, Huang L, Cao P, Shen L, Gui C (2022). Corrigendum: robust rank aggregation and least absolute shrinkage and selection operator analysis of novel gene signatures in dilated cardiomyopathy. Front Cardiovasc Med.

[37] Murayama K, Kawano S. Sparse Bayesian Learning With Weakly Informative Hyperprior and Extended Predictive Information Criterion. *IEEE transactions on neural networks and learning systems* 2021, Pp. 10.1109/TNNLS.2021.3131357 34890342

[38] Liu J, Hao J, Sun Y, Shi Z (2021). Network analysis of population flow among major cities and its influence on COVID-19 transmission in China. Cities (London England).

[39] Ren L, Wang Y, Wu L, Wei Z, Cui LB, Wei X, Hu X, Peng J, Jin Y, Li F (2021). Network structure of depression and anxiety symptoms in chinese female nursing students. BMC Psychiatry.

[40] Ren L, Wei Z, Li Y, Cui LB, Wang Y, Wu L, Wei X, Peng J, Li K, Jin Y (2021). The relations between different components of intolerance of uncertainty and symptoms of generalized anxiety disorder: a network analysis. BMC Psychiatry.

[41] Epskamp S, Borsboom D, Fried EI. Estimating psychological networks and their accuracy: a tutorial paper. Behav Res Methods. 2018;50(1):195–212. 10.3758/s13428-017-0862-1 PMC5809547 28342071

[42] Jones PJ, Ma R, McNally RJ. Bridge centrality: a network approach to understanding comorbidity. Multivar Behav Res. 2019:1–15. 10.1080/00273171.2019.1614898 31179765

[43] Byrne ME, Tanofsky-Kraff M, Lavender JM, Parker MN, Shank LM, Swanson TN, Ramirez E, LeMay-Russell S, Yang SB, Brady SM (2021). Bridging executive function and disinhibited eating among youth: a network analysis. Int J Eat Disord.

[44] Van der Hallen R, Jongerling J, Godor BP (2020). Coping and resilience in adults: a cross-sectional network analysis. Anxiety Stress Coping.

[45] Coelho L, Hauck K, McKenzie K, Copeland JL, Kan IP, Gibb RL, Gonzalez CLR (2020). The association between sedentary behavior and cognitive ability in older adults. Aging Clin Exp Res.

[46] Adam L, O’Connor C, Garcia AC (2018). Evaluating the impact of diabetes self-management education methods on knowledge, Attitudes and Behaviours of adult patients with type 2 diabetes Mellitus. Can J diabetes.

[47] Heller SR, Peyrot M, Oates SK, Taylor AD. Hypoglycemia in patient with type 2 diabetes treated with insulin: it can happen.BMJ open diabetes research & care2020, 8(1). 10.1136/bmjdrc-2020-001194 PMC7299018 32546549

[48] Choi SY, Ko SH (2021). Severe hypoglycemia as a preventable risk factor for cardiovascular disease in patients with type 2 diabetes mellitus. Korean J Intern Med.

[49] Tian K, Li Chang AA, Choudhary P, Xin X, Bee YM, Yen GS, Teh MM (2022). High incidence of undetected low sensor glucose events among elderly patients with type 2 diabetes more than a decade on after the ACCORD study. Curr Med Res Opin.

[50] Silbert R, Salcido-Montenegro A, Rodriguez-Gutierrez R, Katabi A, McCoy RG (2018). Hypoglycemia among patients with type 2 diabetes: epidemiology, risk factors, and Prevention Strategies. Curr Diab Rep.

[51] Khunti K, Gomes MB, Pocock S, Shestakova MV, Pintat S, Fenici P, Hammar N, Medina J (2018). Therapeutic inertia in the treatment of hyperglycaemia in patients with type 2 diabetes: a systematic review. Diabetes Obes Metab.

[52] Ali Abdelhamid Y, Bernjak A, Phillips LK, Summers MJ, Weinel LM, Lange K, Chow E, Kar P, Horowitz M, Heller S (2021). Nocturnal hypoglycemia in patients with diabetes discharged from ICUs: a prospective Two-Center Cohort Study. Crit Care Med.

[53] Siamashvili M, Davis HA, Davis SN (2021). Nocturnal hypoglycemia in type 1 and type 2 diabetes: an update on prevalence, prevention, pathophysiology and patient awareness. Expert Rev Endocrinol metabolism.

[54] Nery C, Moraes SRA, Novaes KA, Bezerra MA, Silveira PVC, Lemos A (2017). Effectiveness of resistance exercise compared to aerobic exercise without insulin therapy in patients with type 2 diabetes mellitus: a meta-analysis. Braz J Phys Ther.

[55] Seyedizadeh SH, Cheragh-Birjandi S, Hamedi Nia MR. The Effects of Combined Exercise Training (Resistance-Aerobic) on Serum Kinesin and Physical Function in Type 2 Diabetes Patients with Diabetic Peripheral Neuropathy (Randomized Controlled Trials). *Journal of diabetes research* 2020, 2020:6978128. 10.1155/2020/6978128 PMC7085367 32215272

[56] Silva JSC, Seguro CS, Naves MMV (2022). Gut microbiota and physical exercise in obesity and diabetes - A systematic review. Nutr metabolism Cardiovasc diseases: NMCD.

